# Breast Carcinoma Receptor Expression in a Caribbean Population

**DOI:** 10.1055/s-0042-1756632

**Published:** 2022-09-19

**Authors:** Michael J. Ramdass, Joshua Gonzales, Dale Maharaj, Donald Simeon, Shaheeba Barrow

**Affiliations:** 1 Department of Clinical Surgical Sciences, The University of the West Indies, St. Augustine, Trinidad and Tobago, West Indies; 2 Department of Surgery, Port of Spain General Hospital, Port of Spain, Trinidad and Tobago; 3 School of Medicine, Royal College of Surgeons in Ireland, Dublin, Ireland; 4 Caribbean Centre for Health Systems Research and Development, The University of the West Indies, St. Augustine, Trinidad and Tobago, West Indies; 5 Department of Pathology, Port of Spain General Hospital, Port of Spain, Trinidad and Tobago

**Keywords:** breast carcinoma, receptors, West Indian, Caribbean, East Indian, Afro-Caribbean

## Abstract

Trinidad and Tobago are islands in the Southern Caribbean with a unique mix of races within the population consisting of East Indian (EI) (37.6%), Afro-Caribbean (AC) (36.3%), mixed (24.2%), and Caucasian, Chinese, Lebanese, Syrian, Amerindian, and Spanish groups accounting for 1.9%. It makes it suitable for a comparison of breast carcinoma receptor expression within a fixed environment. This study included 257 women with an age range of 28 to 93 years (mean = 57.2, standard deviation = 15.0), peak age group of 51 to 60 consisting of 105 EI, 119 AC, and 33 mixed descent. Invasive ductal carcinoma accounted for 88%, invasive lobular 9.7%, and ductal carcinoma in situ 2.3%. The triple-negative rates were 24.8, 33.6, and 30.3% for EI, AC, and mixed races, respectively, with the Pearson's chi-square test revealing statistical significance for the AC versus EI ( *p* < 0.001); AC versus mixed ( *p* < 0.001); and EI versus mixed ( *p* = 0.014) groups. The overall estrogen (ER), progesterone (PR), and human epidermal growth receptor (HER) expression negative rates were 52, 64, and 79%, respectively. Chi-square test of the following combinations: ER +/PR +/HER + ; ER +/PR +/HER − ; ER −/PR −/HER + ; ER +/PR −/HER + ; ER +/PR −/HER − ; ER −/PR +/HER + ; ER −/PR +/HER− revealed no statistical differences ( *p* = 0.689).

Breast cancer is the most common cancer and leading cause of cancer-related mortality in females worldwide. In 2018, there was an estimated 18.1 million new cases and 9.6 million new deaths, with an incident rate of 11.6% worldwide. 1 The highest incidence rates are in Western Europe and the United States and the lowest in developing countries such as Africa and Asia. In the Caribbean and Latin America, breast cancer is frequently diagnosed alongside cervical cancer. 2 In the local population in 2018, the highest incidence rates and mortality for breast cancer were in the 45 to 54 and 55 to 64 year age groups with the most commonly diagnosed cancer in women being breast cancer with a rate of 46.6 per 100,000 population. 3

Breast cancer was traditionally classified according to morphologic features, but we now know that it is heterogenous with a myriad of molecular subtypes. The relatively new and evolving molecular classification uses immunohistochemistry (IHC) to identify receptors including estrogen (ER), progesterone (PR), and human epidermal growth receptor (HER)2/neu expression which are critical for planning targeted treatment regimes. 4 5 6 7 In 2000, Perou et al first described the “molecular portrait” of breast cancer which included luminal A, luminal B, and HER2/neu overexpression, as well as basal-like. 8 Eleven years later, the St. Gallen Consensus 2011 classified breast cancer into four molecular subtypes, luminal A (ER +/PR +/HER2 −/low Ki-67); luminal B (ER +/PR +/HER2 −/+/high K _i_ -67); HER2-overexpression (ER −/PR −/HER2 + ), and triple-negative breast cancers (TNBCs) (ER −/PR −/HER2 − ). 9

Molecular profiling is critical in determining the systemic treatment regime vis-à-vis endocrine treatment for endocrine-responsive tumors and cytotoxic drugs for nonendocrine-responsive tumors. Tumors with HER2/neu overexpression should be treated with trastuzumab. 10 11 12 TNBC tumors are hormone resistant, proliferative, metastatic with a relatively poor prognosis and are treated with chemoradiotherapy. 13 14

While treatment regimes and responses show a high variance with molecular subtyping, demographics appear to have a significant influence on the molecular portrait of the breast cancer patient. Several population-based studies have demonstrated a divergence in molecular subtypes with ethnicity and geography, 15 16 17 18 19 20 and include the United States, 21 China, 22 Africa, 23 and Saudi Arabia. 24

The aim of this study is to determine the molecular profile of the Caribbean female with respect to breast cancer and further, to compare receptor distribution in a primarily biethnic population in a constant environment and socioeconomic background. The receptor expression isolated by our pathology department up to the time of this study included ER, PR, and HER2, all of which play a major role in prognosis and management of the disease among the population of Trinidad and Tobago.

## Patients and Methods

Data were retrospectively collected on all cases of female breast cancer presenting to the Port of Spain General Hospital for the year 2015. Demographic data were collected including age, gender, histologic type, race, and receptor status. Patients were classified into three main ethnic groups including East Indian (EI), Afro-Caribbean (AC), or mixed races with the minority groups (Caucasian, Chinese, Arab, and Spanish) excluded. The receptor expression analyzed included ER, PR, and HER2/neu.

The IHC was performed using the following technique: 4-mm paraffin-embedded sections were prepared and tissue sections were boiled in 10 mM citrate buffer (pH 6.0) for 10 minutes followed by cooling at 25°C. Sections were covered with monoclonal mouse antihuman ER (clone 1D5; Zytomed Systems, Berlin, Germany), monoclonal mouse antihuman PR (clone 636; Dako, Carpinteria, CA), and HercepTest (Dako) for HER2/neu by using a semiautomated system (IntelliPath; Biocare Medical, Pacheco, CA). ER and PR were considered positive if >1% nuclei of tumor cells stained according to the American Society of Clinical Oncology/College of American Pathology guidelines for both the Sudanese and German patients. HER2 was scored as 0, 1 + , 2 + , or 3 + . Fluorescent in situ hybridization was not performed for intermediate 2+ HER2 in both groups; only a score of 3+ was considered HER2 enriched, whereas scores < 2+ were assumed to be HER2 negative. Furthermore, K _i_ -67 was not assessed to evaluate the mitotic index. Subtypes were defined as luminal A (ER− and/or PR positive and HER2 negative), luminal B (ER− and/or PR positive and HER2 positive), HER2 type (ER− and PR negative and HER2 positive), and triple negative.

Permission was granted from the relevant hospital authority and ethics board to collect information from patients' notes and the electronic medical records for research purposes. Data analysis was performed using the SPSS version 24 (SPSS, Chicago, IL). Ethnicity differences in molecular subtypes and demographics were compared using the chi-square test. Differences in mean age were analyzed using analysis of variance. The statistical significance level was set at *p* < 0.05.

## Results

**Fig. 1:**
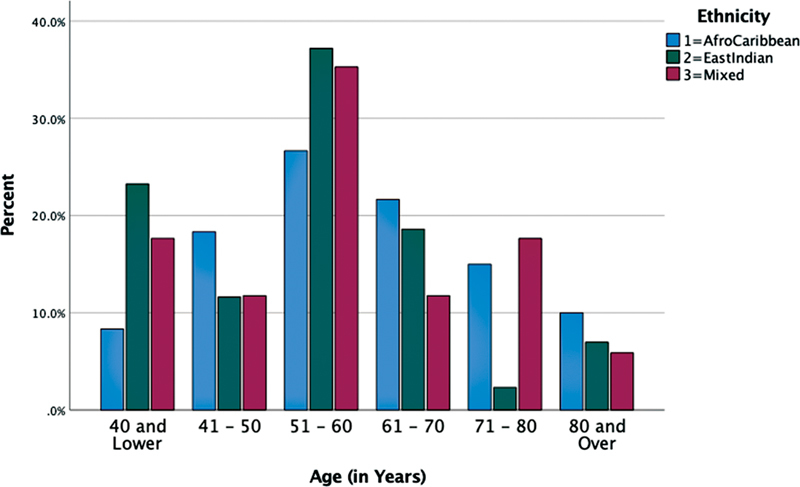
Age distribution, by ethnicity.

There were 257 women with an age range of 28 to 93 years, peak age group of 51 to 60 years (mean = 57.3, standard deviation = 15.0) consisting of 105 EI, 119 AC, and 33 women of mixed race. There was no difference in mean age of presentation by ethnicity ( *p* = 0.142). The age distribution is illustrated in Fig. 1 with comparisons to ethnicity illustrated.

Histologically, 226 (88%) of the tumors found were invasive ductal carcinoma, 25 (9.7%) were invasive lobular carcinoma, and 6 (2.3%) were ductal carcinoma in situ.

The overall triple-negative rate in all races was 29.6% (76/257) and subgroup triple-negative rates were 24.8% (26/105), 33.6% (40/119), and 30.3% (10/33) for EI, AC, and mixed races, respectively, with a statistical difference on Pearson's chi-square test between the following groups: EI versus AC ( *p* < 0.001); AC versus mixed ( *p* < 0.001); and EI versus mixed ( *p* < 0.014). The overall ER, PR, and HER negative rates were 52% (EI), 64%(AC), and 79% (mixed), respectively. Chi-square test of the following combinations: ER +/PR +/HER + ; ER +/PR +/HER − ; ER −/PR −/HER + ; ER +/PR −/HER + ; ER +/PR −/HER − ; ER −/PR +/HER + ; and ER −/PR +/HER− revealed no statistical differences ( *p* = 0.689) ( Table 1 ).

**Table 1:** Comparison of demographic data and receptor expression in dominant ethnic groups

Ethnicity	All patients ( *n* = 257)	EI( *n* = 105)	AC ( *n* = 119)	Mixed ( *n* = 33)	*p* -Value
Age range	28–93	31–93	28–89	31–89	
Mean age (SD)	57.3 (15.0)	53.9 (14.8)	59.8 (14.5)	56.7 (16.4)	0.142
Peak age group	51–60	51–60	51–60	51–60	
Histology					0.964
Invasive ductal	226 (87.9%)	94 (89.5%)	104 (87.4%)	28 (84.8%)	
Invasive lobular	25 (9.7%)	9 (8.6%)	12 (10.1%)	4 (12.1%)	
DCIS	6 (2.3%)	2 (1.9%)	3 (2.5%)	1 (3.0%)	
ER+	123 (47.9%)	47 (44.7%)	58 (48.7%)	18 (54.5%)	0.597
PR+	93 (36.2%)	41 (39.0%)	34% (40)	36% (12)	0.700
HER+	53 (20.6%)	25 (23.8%)	23 (19.3%)	5 (15.2%)	0.502
Receptor expression for all combinations is given below					0.689
ER −/PR −/HER−	29.6% (76)	24.8% (26)	33.6% (40)	30.3% (10)	Triple −ve Pearson's chi-square test EI vs. AC, *p* < 0.001; EI vs. mixed, *p* = 0.014; AC vs. mixed, *p* < 0.001
ER +/PR +/HER+	3.1% (8)	1.9% (2)	4.2% (5)	3.0% (1)	
ER +/PR +/HER−	24.1% (62)	23.8% (25)	22.7% (27)	30.3% (10)	
ER −/PR −/HER+	13.6% (35)	17.1% (18)	10.9% (13)	12.1% (4)	
ER +/PR −/HER+	3.1% (8)	3.8% (4)	3.3% (4)	0	
ER +/PR −/HER−	17.5% (45)	15.2% (16)	18.5% (22)	21.2% (7)	
ER −/PR +/HER+	0.8% (2)	1.0% (1)	0.8% (1)	0	
ER −/PR +/HER−	8.2% (21)	12.4% (13)	5.9% (7)	3.0% (1)	

Abbreviations: AC, Afro-Caribbean; DCIS, ductal carcinoma in situ; EI, East Indian; ER, estrogen; PR, progesterone; SD, standard deviation.

## Discussion

It is evident from studies done locally that breast cancer is one of the leading causes of mortality in women in the Caribbean region as seen from a retrospective analysis of a 35-year period from 1970 to 2004 in Trinidad and Tobago. The general pattern of increase was observed in both crude and age-standardized mortalities. The overall average crude mortality was 15.6 per 100,000 women, and the average age-standardized mortality was 18.0 per 100,000 women. There was a pattern of increase in mortality with increasing age. The mortality rate was considerably higher for the age groups older than 50 years than those younger than 50 years both showing an upward trend over the 35-year period. 25 26 In a study in the eastern part of Trinidad in Sangre Grande, it was reported that the 5-year breast cancer survival rate was 74.3%, and the recurrence-free survival rate was 56.4% for the period 2010 to 2015. 27

Another local study concluded that breast density was an important predictor of newly diagnosed breast cancer in Trinidad and Tobago. 28 Warner et al found notable ancestral differences in survival. Women of EI and mixed ancestry experienced significantly longer survival than those of African ancestry; however, differences in survival by geography were not observed. 29

Camacho-Rivera et al published their study done between 1995 and 2005. Their findings noted that of 2,614 cases, ∼50% were diagnosed between the ages of 45 to 59 years, 12.5% before the age of 40 years, 45% of women were diagnosed at a local stage, and 43% were hormone receptor positive. There were no racial/ethnic differences observed with respect to treatment or survival. 30 This is in stark contrast to our study herein presented where we found only 3.1% of the sample to be triple positive and 24.1% to be ER and PR positive and HER negative. The overall triple-negative rates found in our study was 29.6%. The overall ER, PR, and HER positivity rates were 47.9, 36.2, and 20.6%, respectively, with no statistically significant differences among the three ethnic groups on chi-square test.

## Conclusion

The findings in this study reveal that receptor expression among the EI, AC, and the mixed ethnic groups in a setting of similar environmental and socioeconomic factors in this population showed statistically significant differences as demonstrated in Table 1 . It also showed that the overall triple-negative receptor expression rates were close to 30% of the study sample. We hope that these data add new information to the Caribbean and world data on breast cancer receptor expression and conclude that further funding and research need to be channeled toward genetic and biological factors to improve treatment and survival in the Caribbean region.
